# Case Report: Infantile Ischemic Stroke and Antiphospholipid Antibodies, Description of Four Cases

**DOI:** 10.3389/fped.2020.596386

**Published:** 2020-11-24

**Authors:** Teresa Giani, Angela Mauro, Giovanna Ferrara, Rolando Cimaz

**Affiliations:** ^1^Azienda Ospedaliera Universitaria Meyer, Florence, Italy; ^2^Department of Medical Biotechnology, University of Siena, Siena, Italy; ^3^Emergency Department Santobono-Pausilipon Children's Hospital, Naples, Italy; ^4^Azienda Unità Sanitaria Locale Toscana Centro, Florence, Italy; ^5^Azienda Socio Sanitaria Territoriale G-Pini, Milan, Italy; ^6^Department of Clinical Sciences and Community Health and Research Center for Adult and Pediatric Rheumatic Diseases, University of Milan, Milan, Italy

**Keywords:** APS—antiphospholipid antibody syndrome, perinatal stroke, brain damage, autoimmune disorders, transplacental passage autoantibodies

## Abstract

Antiphospholipid syndrome (APS) is a rare condition in childhood, but even more in the neonatal age. Most neonatal cases are considered a passively acquired autoimmune disease, due to a transplacental passage of maternal antiphospholipid antibodies (aPL) from mothers with primary or secondary APS or, more often, from asymptomatic aPL carriers. Exceedingly unusual is the neonatal *de novo* production of aPL. We present four infants with presumed perinatal stroke in presence of increased and persistent aPL levels, even after 6 months of life, opening the window on a gray zone related to the origin of these antibodies (maternal or neonatal) and on their role in the pathogenesis of stroke.

## Introduction

Anti-phospholipid syndrome (APS), first described in 1986, is an autoimmune disorder characterized by a history of venous or arterial thrombosis and/or of pregnancy morbidity in the presence of persistent anti-phospholipid antibodies (aPL) (1, 2). aPL are a heterogeneous family of autoantibodies including lupus anti-coagulant (LA), anti-cardiolipin antibodies (aCL), and anti-β2-glycoprotein I antibodies (β2GPI) (3). APS can be associated with other autoimmune diseases, mainly with systemic lupus erythematosus, but in many cases can be found in isolation as a primary syndrome (4).

APS commonly develops in adults, especially in young women, while in children it is an unusual condition accounting for < 3% of the cases (5). In the neonatal age APS is exceedingly rare. Most of the cases are considered a passively acquired autoimmune disease (6). Maternal IgG isotype aPL from mothers with primary or secondary APS or, more often, from asymptomatic aPL carriers can pass into the fetal circulation, actively transported through the placenta (7).

Only few cases have been described in literature of *de novo* aPL production in neonatal age. A negative maternal aPL profile, a prolonged persistence of aPL in offspring, IgM isotypes may help in distinguishing between *de novo* and acquired autoimmunity, which would be useful for prognostic evaluation and supporting the treatment choice.

Maternal APS is known to predispose to prematurity, and intrauterine growth restriction, and to increase the risk of thrombosis, thrombocytopenia, and developmental delay in neonates (8).

Acute ischemic stroke, arterial and venous thrombosis have been observed in neonatal APS, but additional prothrombotic risk factors may be crucial to trigger the final pathological event in these situations (9).

We present four infants with persistent levels of aPL, even after 6 months of life, who developed brain stroke, and discuss the possible origin of aPL, and their role in early ischemic stroke.

Table 1 summarizes the main features of these four cases, which are described in detail below.

**Table 1:** Clinical and laboratory data of the 4 infants.

		**Case 1**	**Case 2**	**Case 3**	**Case 4**
Prenatal data	Age of the mother at pregnancy	25	34	30	39
	Previous pregnancy related complications	Absent	Absent	2 miscarriages	Absent
	Maternal aPL	Absent	Absent	Anti-β2GPI IgG/IgM: 10/15 U/ml Anti-CL IgG/IgM: 40/46 U/ml LAC IgG/IgM: absent/absent	Absent
Perinatal data	Pregnancy risk factors	*Abruptio placentae* premature uterine contractions	Gestational diabetes	Absent	Gestational diabetes
	Type of delivery	Vaginal	Vaginal	Cesarean section	Cesarean section
	Gestational Age	38+2 w	38 w	39 w	31+6 w
	APGAR score at I and V minute	9–10	10–10	9–9	6–7
	Birth weight	3,450 g	2,770 g	3,600 g	1,350 g
	Perinatal risk factors	Absent	Absent	Absent	Premature rupture of membranes
	Neonate gender	Male	Female	Female	Female
Children	First PAS manifestations	Jerking movements, hypotonia, partial seizures poor spontaneous movements, divergent strabismus	Left hemiparesis	Right hemiparesis	None
	Timing of first PAS manifestations	10 days	5 months	5 months	Radiological signs of cerebral ischemia during perinatal period
	Brain damage area	Left middle cerebral artery territory	Right middle cerebral artery territory	Fronto-temporal-parietal left regions	Fronto-parietal and occipital right regions
	aPL profile Age at testing	Anti-β2GPI IgG/IgM: 60 U/ml/absent Anti-CL IgG/IgM: 45 U/ml/absent LAC IgG/igM: absent/absent 5 months	Anti-β2GPI IgG/IgM: 120 U/ml/absent Anti-CL IgG/IgM: 20 U/ml/absent LAC IgG/IgM: absent/absent 6 months	Anti-β2GPI IgG/IgM: 100 U/ml/Absent Anti-CL IgG/IgM: 101 U/ml/Absent LAC IgG/IgM: 52.5 U/ml/Absent 9 months	Anti-β2GPI IgG/IgM: 70/35 U/ml Anti-CL IgG/IgM: Absent/absent LAC IgG/IgM: absent/absent 23 months
	Additional pro-thrombotic factors	Heterozygous for FVL mutation Homocysteine: 19 μmol/L	Absent	D-dimer: 314 ng/ml Protein C: 53% Protein S: 39% Plasminogen: 67% Compound heterozygosis for mutations of MTHFR gene	Absent
	Timing of aPL disappearance	9 months	16 months	20 months	26 months
	Treatment	Aspirin (4 mg/kg/d)	None	Aspirin (4 mg/kg/d)	None
	Outcome	Hypotonia, right hemiplegia, delay psychomotor development	Mild neurological impairment	Left hemiparesis	Complete recovery

*aPL, antiphospholipid antibodies; LA, lupus anticoagulant; aCL, anti-cardiolipin antibodies; β2GPI, anti-β2-glycoprotein I antibodies; PAS, perinatal arterial ischemic stroke*.

*Normal values, nv; Anti-β2GPI, IgG <12U/ml; IgM <12 U/ml; Anti-CL, IgG <12 U/ml; IgM <10 U/ml; Homocysteine, <15 μmol/L*.

## Case 1

A baby boy was born at 38 weeks of gestational age via normal vaginal delivery. The mother, a 25-year-old healthy woman, without previous history of miscarriages, had suffered from mild *abruptio placentae* at 11 weeks of gestation and premature uterine contractions at the 30th week. Routine blood tests during pregnancy were normal, and routine ultrasound scans, the last of which performed 6 weeks before delivery, did not reveal fetal complications. There was no family history for coagulation disorders or vascular events.

Newborn appeared in good general conditions, with APGAR score 9/10 at first and fifth minute, respectively, and weight of 3,450 kg. He was discharged at 48 h of life.

At 10 days of life the baby showed a poor feeding, an asymmetry in the movements and posture with a weak use of his right side, and a preferential attitude of the head to be rotated to the left. At 6 weeks of age the child was brought to the emergency room because of jerking movements of his arms and legs lasting 5–10 min. He appeared hypotonic, with asymmetric Moro reflexes, absence of spontaneous movements of the right limbs, and poor visual alertness. During hospitalization he presented a divergent strabismus, and partial seizures lasting 5–10 min. Results from blood samples, including immune and infection profiles and a routine coagulation study, electrocardiography, and echocardiography were normal. A video-electroencephalogram showed continuous background activity with seizure activity mainly on the left hemisphere.

Magnetic resonance imaging (MRI) of the brain documented in the fronto-parietal-temporal left regions a large pluri-cystic area with altered intensity of signal involving the cortico-subcortical tissues, and extending in depth until the wall of the lateral ventricle, and extensive damages at caudate, striatum, internal and external capsule, thalamus, cerebral peduncle of the midbrain and left hemipons, and bulbar pyramid. The angio-MR revealed a narrowing of the left middle cerebral artery.

A neonatal hypoxic ischemic syndrome was postulated, and an antiepileptic treatment was started with seizure control. At 5 months of age an extensive investigation of prothrombotic risk factors was performed, revealing aCL IgG 45 U/ml (n.v. <12) with negative IgM, anti-β2GPI IgG 60 U/ml (n.v. <12) with negative IgM, and homocysteine level of 19 μmol/L (<15). Furthermore, the child resulted heterozygous for factor V Leiden (FVL) mutation, like his father, while his mother was negative.

A neonatal APS related to possible transplacental passage of maternal IgG antibodies was hypothesized, and low dose aspirin was prescribed.

Antibody testing for autoimmune disorders, and thrombophilia panel including aPL were obtained in his mother 6 months after delivery and resulted all negative or normal, except for a positive ANA result (1:160, speckled).

At 9 months of age the aPL were absent in the child, and aspirin prophylaxis was interrupted.

At last follow-up, at 13 months of age, the patient still appeared hypotonic, with an insufficient head control, a right hemiplegia and a global delay in his psychomotor development.

## Case 2

A female was born at 38 weeks of gestation via normal vaginal delivery from a 34-year-old woman, at her first pregnancy. APGAR score was 10/10 at first and fifth minute, respectively, and birth weight was 2,770 kg. She was discharged 48 h later in good general conditions. The pregnancy was complicated by insulin-controlled maternal gestational diabetes. There was no history of miscarriages or family history of coagulation disorders or vascular events.

When she was 5 months old her parents observed a progressive reduction in the use of their child's left arm. On clinical examination hemiparesis of upper and lower left limbs, and microcephaly were noticed. Electroencephalogram showed an extensively unstructured right rhythm, without clear signs of epilepsy. Cerebral MRI and MR angiography, performed at 6 months of life, revealed a right atrophy of the white matter, basal and thalamic ganglia, and encephalic trunk, with wallerian degeneration of corticospinal bundle, evocating an ischemic event involving the right middle cerebral artery territory supposed to have occurred several months before, presumably during the perinatal period.

At 6 months of age routine laboratory tests were repeated including a thrombophilia risk screen. Increased values of anti-β2GPI IgG (120 U/ml), with negative IgM, and of aCL IgG (20 U/ml), with negative IgM were documented.

Autoimmune panel and thrombophilia panel including aPL were also performed in her mother when the child was 10 months old and all resulted negative except for the presence of low titer ANA (1:80).

Three months later the patient's anti-β2GPI IgG were still present at 118 U/ml, while aCL were absent. IgG β2GPI progressively reduced and disappeared by the age of 16 month.

Being aPL persistently negative in the absence of other prothrombotic risks factors no treatment was started. One year later, following a regular rehabilitation program, the patient's neurologic status showed a satisfactory functional recovery.

## Case 3

A female baby was born at term by elective cesarean section for previous cesarean, without prenatal complications or abnormalities. No postnatal resuscitation was required, APGAR score was 9 /9 at first and fifth minute, respectively, and birth weight 3,600 kg.

The mother, a 30-year-old woman with vitiligo, reported 2 previous miscarriages: the first at 9 weeks of gestation, the second at 12 weeks. In this last occasion a screening for aPL was performed revealing aCL IgM 38 U/ml, IgG 30 U/ml, anti-β2GPI IgM 20 U/ml, and IgG 20 U/ml. She denied any previous thrombotic events.

At 5 months of age her parents began to notice a reduced use of her right arm. A neurological evaluation confirmed a right-side hemiparesis.

Blood tests and coagulation studies were ordered including full blood count, urea and electrolytes, liver function tests, coagulation profile, and D-dimer and all resulted within the normal limits.

**Figure 1:**
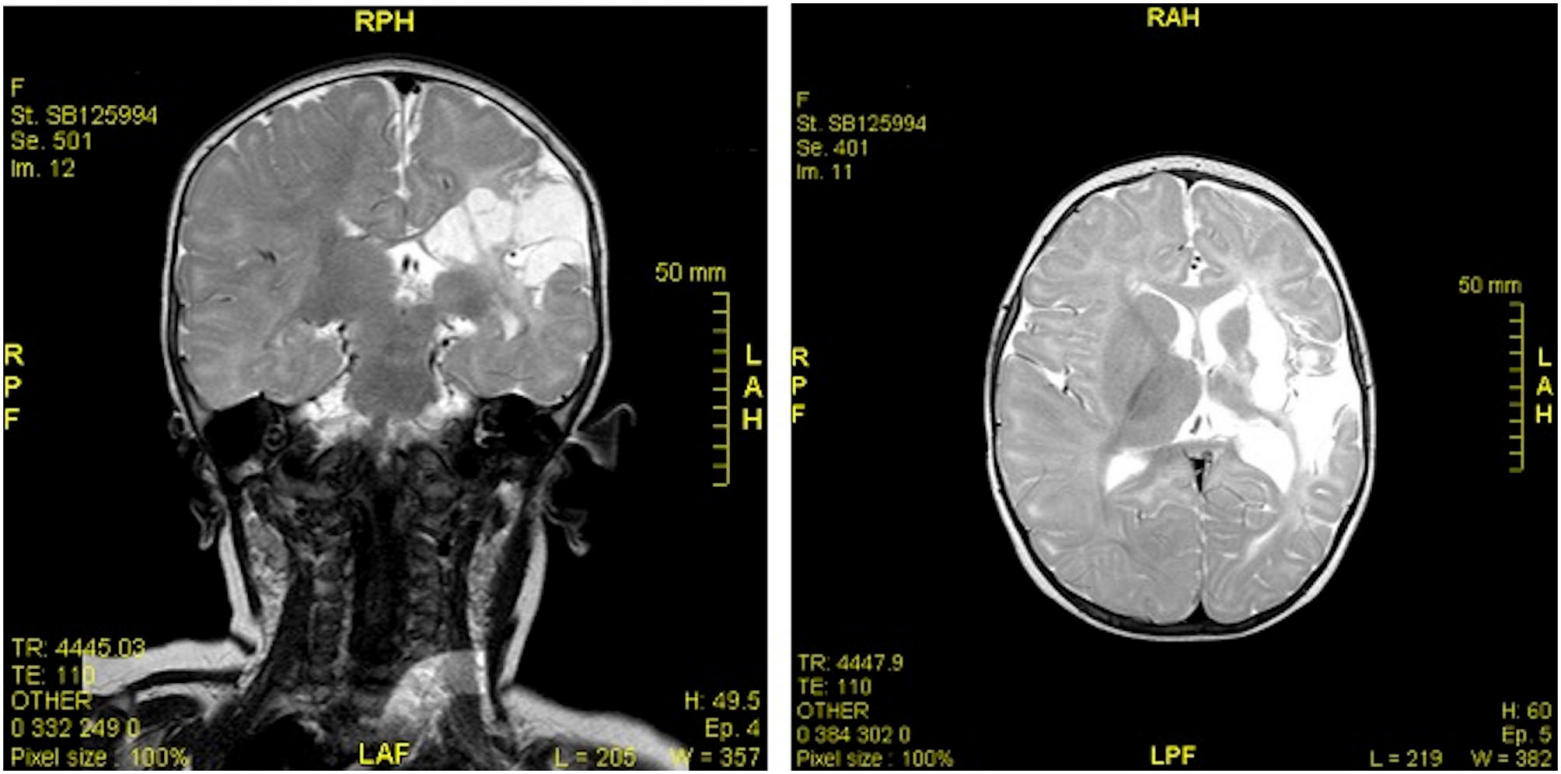
MR imaging (coronal and axial sections) showed ex-vacuo left ventricular dilatation and a cortico-subcortical fronto-temporal-parietal left malacia extending to the ipsilateral capsular regions and to the basal ganglia, without the involvement of the head of the caudate nucleus or the thalamus.

The EEG recorded abnormal left hemisphere electrogenesis. The CT scan identified a cortico-subcortical left fronto-parietal extended malacic area. MR imaging showed ex-vacuo left ventricular dilatation and a cortico-subcortical fronto-temporal-parietal left malacia extending to the ipsilateral capsular regions and to the basal ganglia, without the involvement of the head of the caudate nucleus or the thalamus (Figure 1). A perinatal hypoxic-ischemic damage was supposed, and a physiotherapy treatment was started to promote recovery.

At 9 months of age the child was referred to our hospital for a second opinion. On physical examination, a mild right hemiparesis with decreased muscle strength was observed. A full thrombophilia screen was required and laboratory tests resulted in normal range/negative except for: D-Dimer (314 ng/ml, normal range <250), protein C (53%, normal range 70–140), protein S (39%, normal range 54–124), plasminogen (67%, normal range 80–120), aCL IgG 101 U/ml, IgM negative, anti-β2GPI IgG >100 U/ml, and IgM negative. A compound heterozygosis for the C677T and A1298C mutations of the methylenetetrahydrofolate reductase (MTHFR) gene was detected. Mother's serological examinations, performed when the child was 10 months old, showed aCL IgG 40 U/ml, IgM 46 U/ml, and anti-β2GPI IgG 10 U/ml, IgM 15 U/ml.

Treatment with baby aspirin was prescribed, and a physical and occupational therapy program were recommended. aPL were periodically checked and disappeared after 20 months of age. At last follow-up, at the age of 3 years, the child appeared in good general conditions with persistent, mild neurologic outcome.

## Case 4

A 22-month-old female infant presented to our attention for a 1-month history of skin color changes. The distal extremities of her hands, sometimes also of her feet and tongue, dramatically changed from white to blue discoloration upon exposure to cold.

The baby was born from a dichorionic-diamniotic twin pregnancy complicated at the 25th week with gestational diabetes. The mother was a previously healthy 39-year-old primigravida. She was the first-born twin after an emergency cesarean section, required at the 31st week due to uterine contractions and rupture of membranes. The APGAR score was 6 and 7 at first and fifth minutes, respectively; birth weight was 1,350 g. She received resuscitation procedures, including intubation because of respiratory distress and bradycardia. She was admitted to the neonatal intensive care unit where she received surfactant, and mechanical ventilation, then needed oxygen for 21 days.

She was discharged about 3 months later, but despite a normal neurological exam and an adequate psychomotor development she underwent several cerebral echographies in the first year of life due to her prematurity. White matter hyperechogenicity in the periventricular and subcortical area of the right parietal lobe, and bilateral periventricular hyperechoic zones at the occipital lobe were detected.

**Figure 2:**
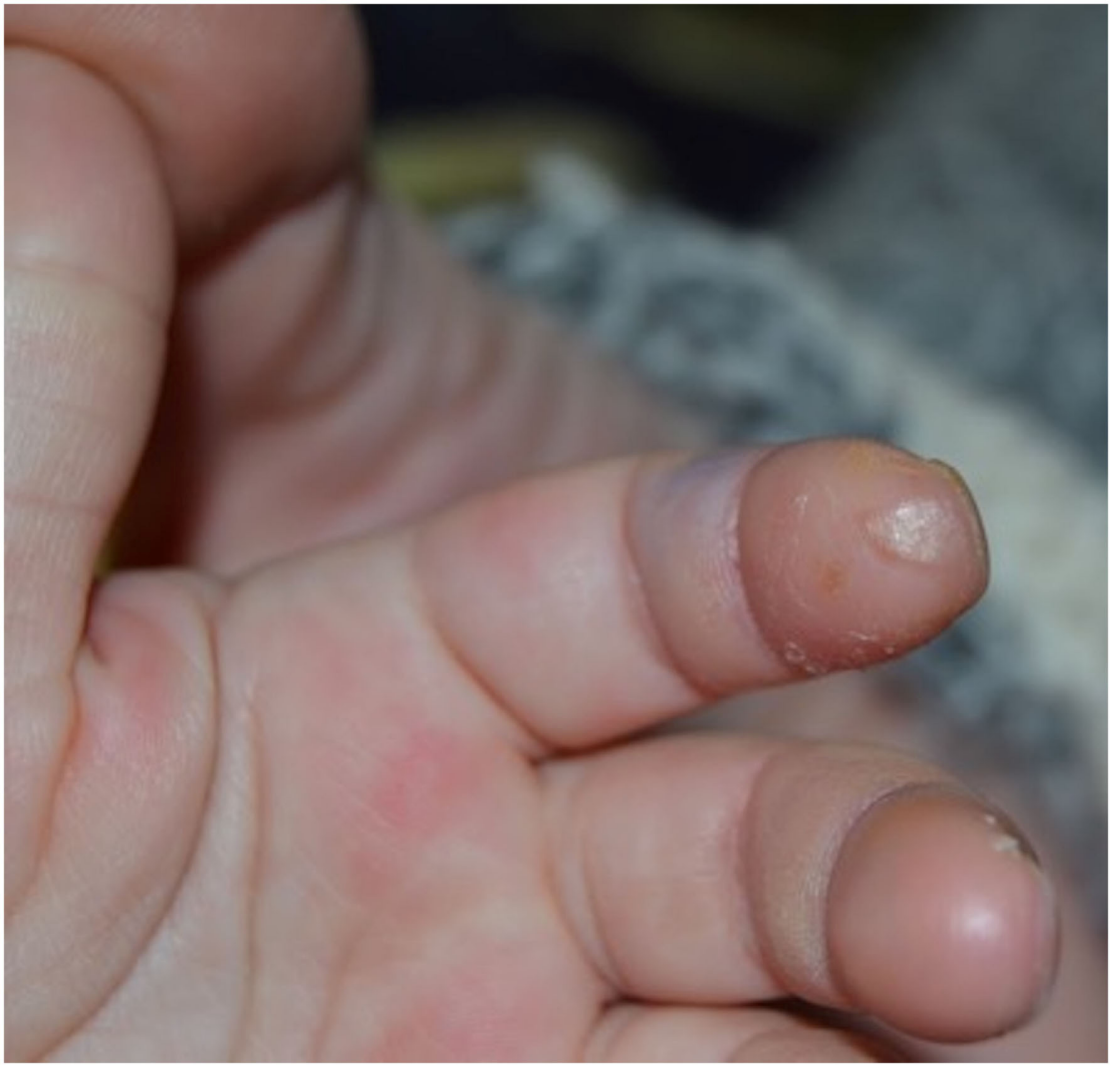
White scar over the tip of the second finger of the right hand derived from a long-lasting ulcerative lesion.

When she came to our attention she appeared in good general conditions with a normal neurological exam. A white pitting scar over the tip of the second finger of the right hand was observed (Figure 2). Screening investigations for inherited thrombophilia revealed anti-β2GPI IgG 70 U/ml, and IgM 35 U/ml.

MRI showed a cystic encephalomalacia at the right frontal circumference of the cortical-subcortical site with cortical thinning, mild ventricular dilatation, mild white matter damage near the corona radiata and in the periventricular frontal area.

Mother was promptly tested for autoimmunity, with negative results including aPL. We followed the baby for 2 years. Acrocyanosis gradually resolved within 6 months from the first visit, and anti-β2GPI antibodies disappeared, at 26 months of age.

## Discussion

Ischemic stroke has been reported to be the presenting condition in more than a quarter of pediatric patients with APS, as resulted from a multi-national childhood APS registry comprising 121 subjects, including both primary and secondary forms. However, the real impact of APS in the neonatal stroke is unclear (5).

On the other hand, cerebral stroke in neonatal-perinatal period encompasses heterogeneous conditions leading to focal neurological injuries (10). Perinatal arterial ischemic stroke (PAS) is defined as a focal (or multifocal) cerebral ischemic infarction, confirmed by radiological or pathological assessment, due to an arterial event, and occurring between 28 weeks' gestation and 28 days of postnatal age. The incidence of perinatal stroke has been estimated at about 1:3,000–4,000 live births (11).

Perinatal stroke may present acutely in the first week of life with clear neurological symptoms such as seizures, lethargy, apnea and/or poor feeding. However, around 40% of cases are clinically asymptomatic until several months of age, the so called “presumed perinatal strokes” (12–14).

Almost half of perinatal strokes cause a neurological deficit. PAS is the main event responsible for severe outcomes such as neurological disabilities, epilepsy, cognitive impairment (15).

The pathophysiology of perinatal stroke is complex and includes different, possible causes. Risk factors may be related both to maternal and placental conditions, as well as to fetal and neonatal disorders. Maternal preeclampsia, chorioamnionitis, fetal cardiac anomalies, polycythemia, birth asphyxia, and systemic infection are typically described in PAS.

The way genetic and acquired thrombophilic factors act in causing PAS is quite controversial and not completely understood (16).

Simchen et al. (17) investigated the role of maternal and infant prothrombotic factors in a cohort of infants with a history of PAS and their mothers, concluding that both maternal and infant thrombophilia may predispose to PAS. In this study at least one thrombophilic marker was documented in 64% of children, and among 68% of mothers. FVL, protein C deficiency, and aPL prevailed among infants, in particular, more than one-fifth of PAS infants resulted positive for aPL, compared to 5.4% of controls. Almost one-third of mothers resulted positive for FVL, and 18.2% them had aPL (vs. 4.7% of control mothers) (17).

Only part of newborns born to aPL-positive women really acquire aPL antibodies. Data from the European neonatal registry regarding 141 babies born to aPL-positive mothers revealed a low transmission rate (20, 25 and 43%, respectively, for LA, aCL and anti-β2GP1) and no cases of PAS This is in part linked to a filtering effect exerted by placenta through the β2GP1 on trophoblasts membrane, that significantly reduces maternal antibodies transfer (18).

After birth, transmitted antibodies usually disappear within the first months of life (19).

However, also in presence of neonatal antibodies the related manifestations are exceedingly rare, being aPL unable to induce a thrombotic process in the absence of an additional thrombophilic factors (20). Hereditary and/or acquired prothrombotic conditions such as asphyxia, sepsis, dehydration, arterial and venous catheters may contribute to trigger the ischemic event. Therefore, aPL act as a predisposing factor for PAS, in presence of which a thrombophilic screening should be considered (21).

We described four cases of presumed perinatal strokes, as all babies seemed neurologically normal in the neonatal period and up to 28 days post-partum.

Three out of the four children we described (cases 1, 2, and 3) had aPL IgG isotypes, but only one of the paired mothers tested positive (for both aCL and anti-β2GP1). The other two women resulted negative but were tested 6 months after birth, so a transient aPL positivity during pregnancy cannot be excluded. In these children aPL disappeared within 2 years of life (at 6, 16 e 20 months, respectively); we hypothesize an acquired APS due to transplacental autoantibody passage on the basis of the depletion of IgG aPL, and the absence of IgM antibodies. In two of these subjects a predisposing prothrombotic risk factor, as the heterozygosity for factor V Leiden in case 1, and low levels of protein C and S combined to the compound heterozygosity for MTHFR in case 3, had been identified. The exact timing of the ischemic event, as well as the significance of *abruptio placentae* and preterm contractions as predisposing prothrombotic conditions in case 1 remains uncertain.

Case 4 clearly shows a predisposing condition for PAS, being a preterm twin with a perinatal history of resuscitation and ventilation. This baby presented IgM isotypes of anti-β2GP1 antibodies, and her mother tested negative for aPL suggesting a *de novo* APS type. Distinguishing maternally transmitted from *de novo* neonatal APS may be helpful to define the prognosis and to manage the treatment and the follow-up. *De novo* APS may be associated with signs and symptoms (22), such as thrombocytopenia and skin manifestations (i.e., livedo reticularis and Raynaud's phenomenon), which we observed in our case 4. We think that also peripheral ischemia and ulcerative lesions were favored by the thrombophilia due to the presence of aPL, and indeed with the disappearance of these antibodies skin manifestations did not recur.

Most of the neonatal *de novo* APS cases present a multiple aPL positivity, and have a prolonged persistence of antibody titers, while in the acquired type antibodies decline and disappear by 6–12 months (23–25).

Diagnosis of *de novo* neonatal APS is often delayed with a mean time of 4.7 months, probably due to a low awareness of this condition among clinicians, while maternally transmitted APS has been shown to be identified earlier (26).

At the present time, it is still unclear whether outcomes for *de novo* and transmitted neonatal APS are different, and whether the *de novo* APS may precede other autoimmune diseases, such as systemic lupus erythematosus (26–34).

Being a retrospective case series, our report has some limitations: the timing of aPL testing in mother and infant varied and was not followed at same intervals; moreover, none of the children had testing completed at the time of the stroke since they became symptomatic several months after the event. However, pediatric APS is rare and data is lacking in neonatal APS, particularly in cases that may represent primary APS. Our paper is unique in that these infants' mothers were otherwise healthy without known APS or rheumatologic disease and we found elevated antiphospholipid antibodies in the children only since we looked for them.

In conclusion, neonatal APS is a rare hypercoagulation disorder that can predispose to early acute ischemic stroke. The detection of aPL has to be included in the thrombophilic screening in infants who experience a cerebral event, especially when the timing of the stroke is not known like in our cases, even in the absence of a maternal history suggestive for APS or autoimmune disorders. Screening for additional congenital and acquired prothrombotic risk factors should also always be performed in such cases as recommended in the last guidelines for the management of stroke in children (35). The absence of antibodies in the mothers does not rule out the diagnosis of APS in a newborn, since even if rare a *de novo* production of autoantibodies cannot be excluded.

## Data Availability Statement

The original contributions presented in the study are included in the article/supplementary materials, further inquiries can be directed to the corresponding author/s.

## Author Contributions

All authors contributing to the writing of the manuscript, critical review, and accepted the final version.

## Conflict of Interest

The authors declare that the research was conducted in the absence of any commercial or financial relationships that could be construed as a potential conflict of interest.
